# A comprehensive review of the clinical progress of esketamine: From anesthesia to antidepressant therapy

**DOI:** 10.1097/MD.0000000000048570

**Published:** 2026-05-01

**Authors:** Hai-lin Hu, Qi-zhou Huang, Ping-xia Xie

**Affiliations:** a Department of Guizhou Medical University, Guiyang, Guizhou, China; b Department of Guizhou Provincial People’s Hospital, Guiyang, Guizhou, China.

**Keywords:** anesthesia, antidepressant, depression, Esketamine, NMDA receptor, suicidal ideation, treatment-resistant depression

## Abstract

Esketamine, the S-enantiomer of ketamine, has emerged as a rapid-acting antidepressant with unique mechanisms. This narrative review synthesizes current evidence on its clinical applications, safety, and regulatory status based on peer-reviewed literature published between January 2000 and March 2024, searched in PubMed, Web of Science, and the Cochrane Library. Esketamine exerts its effects primarily through noncompetitive antagonism of N-methyl-D-aspartate receptors, leading to rapid modulation of glutamatergic signaling and neuroplasticity. In anesthesia, it provides effective sedation with minimal respiratory depression. In psychiatry, intravenous and intranasal esketamine have demonstrated rapid antidepressant effects in treatment-resistant depression, with response rates of 50% to 70% within 24 hours. However, long-term safety data remain limited, and concerns persist regarding dissociative symptoms, cognitive impairment, and abuse potential. Regulatory approvals vary: the Food and Drug Administration approved intranasal esketamine for treatment-resistant depression in 2019, while European and Asian countries have adopted differing restrictions. Esketamine represents a paradigm shift in depression treatment, but its use requires careful patient selection, monitoring, and risk management. Future research should focus on head-to-head comparisons with other rapid-acting interventions, long-term outcomes, and integration into stepped-care models.

## 1. Introduction

Esketamine, a noncompetitive N-methyl-D-aspartate (NMDA) receptor antagonist and the S-enantiomer of ketamine, has demonstrated unique therapeutic value across multiple medical fields since its initial synthesis in the 1960s. Originally developed as an anesthetic agent due to its rapid onset, potent analgesic effects, fast metabolism, and favorable safety profile, it has been widely employed for short surgical procedures and examinations.^[1]^ In recent years, with the rapid advancement of psychiatry and neuroscience, the potential of esketamine in antidepressant treatment has been gradually unveiled, particularly for patients with treatment-resistant depression (TRD), demonstrating significant therapeutic effects.^[2]^ Nevertheless, subsequent controversy has emerged regarding its potential side effects and risk of abuse.^[3]^

To comprehensively assess the clinical utility of esketamine, extensive trials and clinical studies are necessary. This review systematically examines: the drug’s developmental history and regulatory milestones; its chemical structure and mechanisms of action; therapeutic applications in anesthesia and psychiatry, with critical appraisal of evidence from randomized controlled trials (RCTs), observational studies, and meta-analyses; safety profile including long-term outcomes and abuse potential; patient selection, contraindications, and monitoring requirements; and future research directions and unresolved questions.

## 2. Methods

This narrative review was conducted following a systematic literature search. We searched PubMed, Web of Science, and the Cochrane Library for articles published from January 1, 2000, to March 31, 2024. The search strategy combined the terms “esketamine,” “ketamine enantiomer,” “depression,” “treatment-resistant depression,” “suicidal ideation,” “anesthesia,” “safety,” “adverse effects,” and “regulatory approval.”

Inclusion criteria were: original research articles, systematic reviews, or meta-analyses; studies involving human subjects; articles published in English. We excluded case reports, conference abstracts, preclinical studies, and non-English publications. Reference lists of included articles were manually searched for additional relevant studies. A total of 39 articles were included in this synthesis.

As this is a review of published literature, no ethical approval or patient consent was required.

## 3. History and current status of esketamine research

The discovery and application of esketamine have undergone a lengthy and innovative journey. Since its first synthesis in 1962,^[4]^ research and application of esketamine have transitioned from an anesthetic to a treatment for psychiatric disorders, showcasing its broad pharmacological effects. Initially, esketamine was rapidly adopted in clinical settings due to its unique anesthetic properties, especially in surgical anesthesia, pain management, and military medical care.

Entering the 21st century, with in-depth exploration of treatment methods for psychiatric disorders, esketamine has demonstrated significant potential in antidepressant therapy, particularly in providing rapid symptom relief for TRD. This has spurred research into its mechanisms of action, revealing esketamine’s role in modulating neurotransmitter systems and promoting neuroplasticity.^[5]^ Globally, particularly in the United States, Europe, and Asia, basic and clinical research on esketamine continues to advance, leading to its recognition as a treatment option for psychiatric disorders.

Notably, in 2019, the United States Food and Drug Administration (FDA) approved a nasal spray formulation based on esketamine (Spravato, Janssen Pharmaceuticals, Inc. ) for the treatment of depression,^[6]^ marking a significant milestone in its use as a psychiatric treatment. However, as clinical applications expand, ensuring the safety and appropriateness of its use has become a key area of research. Furthermore, additional studies are needed to verify the mechanisms of action, optimal treatment protocols, and long-term effects of esketamine.

## 4. Chemical structure and mechanisms of action of esketamine

The chemical structure and mechanisms of action of esketamine offer various clinical applications. Esketamine is the S-enantiomer of ketamine and possesses a complex chemical structure. It is derived from cyclopentyl-cyclopropanone and contains an important chlorinated phenyl ring in its molecular structure, which is critical for its pharmacological activity. The specific structural arrangement in the esketamine molecule provides high affinity for biomolecules, particularly receptors and ion channels. This molecular affinity is rooted in esketamine’s ability to bind to NMDA receptors in the brain through a specific spatial configuration, resulting in its unique pharmacological effects.^[7]^

The mechanisms of action of esketamine are complex and varied, but its primary effect is believed to result from the noncompetitive inhibition of NMDA receptors in the central nervous system. NMDA receptors are specialized ion channel receptors that are crucial for regulating the signaling of the neurotransmitter glutamate and communication between neurons. By blocking these receptors, esketamine can slow or alter neuronal excitability, which elucidates its effectiveness in providing anesthesia and sedation.^[8]^

In addition to its effects on NMDA receptors, esketamine interacts with other neurotransmitter systems, including dopamine, serotonin, and sigma receptors.^[9]^ These interactions contribute to esketamine’s potential effects in antidepressant and neuroprotective contexts. Especially in terms of its antidepressant effects, esketamine can rapidly elevate levels of brain-derived neurotrophic factor and promote neuroplasticity, providing a new therapeutic mechanism for TRD.^[10]^ As research progresses, scientists are gradually uncovering more molecular-level mechanisms of esketamine, including its modulatory effects on neuroinflammatory responses and its promotion of neuronal regeneration and repair.^[11]^ These discoveries not only deepen our understanding of esketamine’s actions but also establish a new theoretical foundation for its application in neurodegenerative and psychiatric disorders.

## 5. Applications of esketamine in anesthesia and sedation

Esketamine was initially utilized as a rapid onset, short-acting anesthetic in clinical practice. Its introduction has provided significant therapeutic options for surgical anesthesia, acute pain management, and critical care. One of the unique aspects of esketamine is its ability to induce what is termed “dissociative anesthesia,” wherein patients experience a state between wakefulness and sleep while remaining insensitive to pain.^[12]^

Esketamine is characterized by rapid onset, quick elimination, and minimal side effects, along with strong analgesic properties, making it suitable for anesthesia and analgesia in various clinical procedures and minor examinations. As esketamine achieves the required anesthetic potency at only half the dose compared to ketamine, a sub-anesthetic dosage regimen of 0.25 mg/kg is often employed in clinical practice.^[13]^ Its high bioavailability and short elimination half life promote swift recovery post-surgery with reduced incidence of adverse effects, which is highly beneficial for clinical anesthesia.

When used as an anesthetic, esketamine has relatively mild effects on the respiratory and cardiovascular systems and can even, to some extent, increase blood pressure and heart rate, making it particularly valuable in treating patients in shock or those requiring avoidance of hypotension.^[14]^ Furthermore, esketamine exhibits potent analgesic properties. In the management of acute pain, esketamine has proven effective in alleviating pain, particularly in cases of fractures, burns, and post-traumatic pain^[15]^ However, due to the potential for adverse psychological reactions and risk of misuse, strict control of dosage, close monitoring, and patient management are essential to minimize the risk of adverse effects.

## 6. Antidepressant applications: evidence from different study designs

Esketamine exerts its antidepressant effects through mechanisms distinct from traditional antidepressant medications. By non-competitively inhibiting NMDA receptors, it rapidly enhances glutamatergic signaling and promotes synaptogenesis.^[16]^ The evidence supporting its efficacy comes from multiple study types, each with different strengths and limitations.

### 6.1. RCTs

Several phase III RCTs have established the efficacy of intranasal esketamine in TRD. In the TRANSFORM-2 study, Daly et al^[17]^ reported that esketamine plus an oral antidepressant led to a significant reduction in Montgomery-Åsberg Depression Rating Scale score at 28 days compared with placebo plus antidepressant (least squares mean difference −4.0, 95% CI −7.3–−0.6). Similarly, the SUSTAIN-2 long-term study demonstrated sustained efficacy with continued treatment.^[18]^ However, these trials have been criticized for their short duration and potential unblinding due to dissociative effects.

Zarate et al^[19]^ also reported significant rapid antidepressant effects from intravenous esketamine, with good tolerance and efficacy rates of 50.0% at 24 hours, 48.2% at 72 hours, and 43.7% at 7 days. This not only provides immediate relief for patients but also offers potential for acute management and reduction of suicide risk.

### 6.2. Real-world observational studies

Observational studies provide complementary evidence on effectiveness in routine clinical populations. A large European multicenter cohort by Martinotti et al^[20]^ including 218 patients with TRD found that 54% responded to esketamine nasal spray after 1 month, with response sustained at 3 months. These real-world data support the generalizability of RCT findings, though lack of control groups limits causal inference.

Observations have noted that the nasal spray formulation of esketamine can rapidly improve depressive symptoms, consistent with findings from intravenous studies, showing a clear dose-response relationship concerning the improvement of depression symptoms.^[21]^ The nasal delivery route is more convenient and safer than intravenous administration; thus, esketamine nasal spray is becoming a focal point of future antidepressant research.^[22]^

### 6.3. Meta-analyses and post hoc analyses

A meta-analysis by Papadimitropoulou et al^[23]^ pooled data from 4 RCTs and confirmed that esketamine plus antidepressant is superior to antidepressant alone (standardized mean difference −0.32, 95% CI −0.48–−0.16). Post hoc analyses of the TRANSFORM studies have explored predictors of response, suggesting that patients with more severe baseline depression and those with a history of suicide attempts may derive greater benefit.^[24]^

### 6.4. Comparison with other rapid-acting interventions

Head-to-head comparisons between esketamine and other rapid-acting treatments such as intravenous ketamine or electroconvulsive therapy are limited. A network meta-analysis by Hock et al^[25]^ suggested comparable efficacy between esketamine and ketamine, but with different adverse effect profiles. The combination of esketamine with modified electroconvulsive therapy has been explored in small studies,^[26]^ with mixed results; some indicate that esketamine may lower seizure threshold and improve cognitive outcomes, but larger trials are needed.

Based on these positive research outcomes, the FDA approved a nasal spray formulation based on esketamine for the treatment of major depressive disorder in 2019, marking a milestone advancement for esketamine in the realm of antidepressant therapy.^[6]^ Despite the notable therapeutic potential of esketamine, further research and evaluation concerning its potential for misuse and the safety of long-term use are critical.

## 7. Safety profile: long-term outcomes and risk management

While esketamine’s short-term safety is well characterized, long-term data are still accumulating. Key concerns include:

### 7.1. Dissociative symptoms and psychotomimetic effects

Dissociation and perceptual disturbances occur frequently during esketamine administration, typically resolving within hours. A meta-analysis by Smith-Apeldoorn et al^[27]^ reported that 40–60% of patients experience transient dissociative symptoms, which are dose-dependent and more common with intravenous administration. These effects rarely lead to treatment discontinuation but require monitoring and patient reassurance.

### 7.2. Cognitive function

Concerns about potential cognitive impairment arise from ketamine’s known effects on memory. However, studies specifically examining esketamine have shown mixed results. A 6-month open-label extension study^[28]^ found no significant decline in cognitive performance, while a smaller study suggested subtle impairments in verbal memory.^[29]^ Longer-term follow-up is needed, especially in patients receiving maintenance treatment.

### 7.3. Tolerance and dependence risk

As a glutamate receptor modulator with abuse potential, esketamine carries a risk of tolerance and dependence. The FDA requires a Risk Evaluation And Mitigation Strategy for esketamine nasal spray, including patient enrollment and monitoring. Epidemiological data from post-marketing surveillance suggest low rates of misuse when administered under supervision,^[30]^ but real-world evidence remains limited. Patients with a history of substance abuse are generally excluded from trials and may be at higher risk.^[31]^

### 7.4. Long-term physical health effects

Potential effects on urinary tract, hepatobiliary system, and cardiovascular function have been extrapolated from chronic ketamine use. While esketamine appears safer at antidepressant doses, cases of cystitis and liver enzyme elevations have been reported.^[32]^ Regular monitoring of renal and hepatic function is recommended in long-term treatment.

## 8. Patient selection, contraindications, and monitoring

Appropriate patient selection is crucial to maximize benefit and minimize harm. Based on current guidelines,^[33]^ candidates for esketamine therapy should meet the following criteria: diagnosis of TRD (failure of at least 2 adequate antidepressant trials); absence of contraindications such as uncontrolled hypertension, aneurysmal vascular disease, or hypersensitivity to esketamine; no current substance use disorder; capacity to provide informed consent and adhere to monitoring.

Contraindications include: history of psychotic disorder, unstable medical conditions (e.g., recent myocardial infarction, intracranial hemorrhage), and pregnancy/lactation due to insufficient safety data.^[34]^

Monitoring requirements during and after administration include blood pressure measurements (at 40 minutes and 2 hours post-dose), observation for dissociative symptoms, and assessment of suicidal ideation. Long-term monitoring should include periodic cognitive assessment, urine toxicology screening, and evaluation of treatment response.^[35]^

## 9. Regulatory status and guideline context across regions

Approval and accessibility of esketamine vary considerably worldwide. In the United States, the FDA approved intranasal esketamine (Spravato) in March 2019 for TRD in conjunction with an oral antidepressant, and subsequently for major depressive disorder with acute suicidal ideation or behavior.^[6]^ The approval requires administration in a certified healthcare setting with post-dose observation.

In Europe, the European Medicines Agency granted marketing authorization in 2019 for the same indications, but individual member states impose additional restrictions; for example, some countries require prior authorization or limit use to specialized centers.^[36]^

In Asia, approval status is heterogeneous. Japan approved esketamine for TRD in 2020, while China’s National Medical Products Administration approved it in 2023 for TRD under strict hospital-based protocols.^[37]^ Other countries, such as India and South Korea, are in various stages of review.

Clinical practice guidelines have been issued by several professional bodies. The Canadian Network for Mood and Anxiety Treatments recommends esketamine as a third-line treatment for TRD,^[38]^ while the United Kingdom’s National Institute for Health and Care Excellence recommends it only within research settings due to cost-effectiveness concerns.^[39]^

## 10. Future directions and research gaps

Despite significant progress, several key questions remain unanswered:

Head-to-head comparisons: Direct comparisons between esketamine and other rapid-acting interventions (e.g., intravenous ketamine, brexanolone, psychedelic-assisted therapy) are needed to inform treatment algorithms.

Long-term efficacy and safety: Extended follow-up studies (> 1 year) are required to assess durability of response, cognitive effects, and risk of tolerance or dependence.

Predictive biomarkers: Identification of clinical or biological predictors of response could enable personalized treatment strategies.

Integration into stepped-care models: Research is needed to determine the optimal placement of esketamine within depression treatment pathways, including its role relative to electroconvulsive therapy and transcranial magnetic stimulation.

Pediatric and geriatric populations: Safety and efficacy in adolescents and older adults are understudied; dedicated trials are warranted.

Novel formulations and analogues: Development of oral or longer-acting formulations may improve accessibility and adherence.

## 11. Conclusions

Esketamine represents a significant advancement in the treatment of depression, offering rapid symptom relief for patients who have not responded to conventional therapies. Its journey from anesthetic to antidepressant underscores the value of drug repurposing. However, its use must be accompanied by rigorous patient selection, monitoring, and risk mitigation. As evidence accumulates and guidelines evolve, esketamine is poised to become an integral component of personalized psychiatric care.

## Author contributions

**Conceptualization:** Hai-lin Hu.

**Methodology:** Hai-lin Hu, Qi-zhou Huang.

**Supervision:** Ping-xia Xie.

**Validation:** Ping-xia Xie.

**Writing – original draft:** Hai-lin Hu.

**Writing – review & editing:** Ping-xia Xie.
